# Facile detection of botulinum neurotoxin using LSPR nanosensor based on Langmuir–Blodgett films of gold nanoparticles

**DOI:** 10.1039/d3ra05386e

**Published:** 2023-10-24

**Authors:** Nguyen Thanh Huong, Ly Tan Nhiem

**Affiliations:** a Center for Biomedical Analysis Laboratories and Scientific Technical Services- Pasteur Institute in Ho Chi Minh City 167 Pasteur Street, Vo Thi Sau Ward, District 3 Ho Chi Minh City Vietnam; b Faculty of Chemical and Food Technology, Ho Chi Minh City University of Technology and Education 01 Vo Van Ngan Street, Linh Chieu Ward, Thu Duc City Ho Chi Minh City Vietnam nhiemlt@hcmute.edu.vn

## Abstract

In this exploratory study, Langmuir–Blodgett (LB) films of gold nanoparticles (Au NPs) were utilized for the first time to detect botulinum neurotoxin (BoNT) based on localized surface plasmon resonance (LSPR), acting as biosensors. Monolayers of Au NPs were initially transferred onto a transparent polymer substrate using the LB technique. This substrate was then used as the base material for subsequent depositions of capping ligands, and eventually, the BoNT at different concentrations. Upon each deposition, LSPR signals were recorded employing UV-Vis spectroscopy. As a result, it was demonstrated that the LB films transferred at a surface pressure of 35 mN m^−1^ were the optimal choice, capable of detecting BoNT at a concentration as low as 1 pg ml^−1^. Furthermore, it was discovered that the formation of Au NP clusters reduced the sensing capacity of the LB films. This sensor offers advantages such as easy fabrication and a quick detection process that utilizes visible light.

## Abstract 2

In this exploratory study, Langmuir–Blodgett (LB) films of gold nanoparticles (Au NPs) were utilized for the first time to detect botulinum neurotoxin (BoNT) based on localized surface plasmon resonance (LSPR), acting as biosensors.

## Graphical abstract

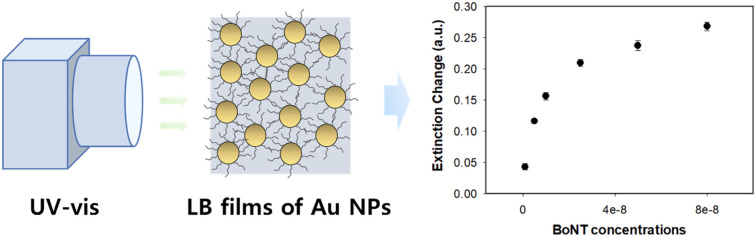

## Introduction

Clostridium botulinum is a bacterium that produces botulinum neurotoxin (BoNT) under low-oxygen conditions, causing respiratory and muscular paralysis.^1,2^ Among the various types of botulism, foodborne botulism is the most common. It is typically caused by the consumption of improperly prepared homemade and/or fermented foods, including meat, fish, and vegetables. This disease can be fatal if not detected and treated with antitoxin; a lethal dose in mice of 1–5 ng kg^−1^ has been recorded.^3^ Therefore, monitoring food samples for BoNT with high accuracy is crucial in preventing botulism. Numerous approaches have been developed to rapidly and accurately detect BoNT. *In vivo* testing is one of the most conventional and effective methods.^4,5^ However, the mouse lethality bioassay, one of the most common methods, is considered inhumane due to animal distress and requires laboratory testing, which may be inaccessible in remote areas. As a result, the non-lethal mouse flaccid paralysis assay has been introduced as an ex vivo approach, which is more economical and humane.

Additionally, *in vitro* methods such as the SNAP-25 assay and immunoassay (ELISA) have been developed.^6,7^ ELISA provides low-cost, rapid detection, and portable tools. However, ELISA-based testing is considered qualitative and has relatively low sensitivity.

Gold nanoparticles (Au NPs), which have recently become one of the most widely used nanomaterials, possess exceptional size-dependent properties.^8,9^ They have been employed in various applications, including diagnostics,^8^ drug delivery,^10^ catalysts,^11^ and most importantly BoNT sensing.^12–14^ The abundance of delocalized electrons moving on the surface, coupled with the presence of an evanescent electric field, makes Au NPs particularly suitable for bio-sensing.^15,16^ This electric field is ultra-sensitive to environmental stimuli upon deposition on the surface and can be easily manipulated by an external electromagnetic field. This interaction is based on a phenomenon known as localized surface plasmon resonance (LSPR). Changes in the surrounding environment can then be monitored by recording shifts in the LSPR wavelength and/or changes in extinction. Notably, UV-vis spectroscopy can be employed to record these LSPR signals. Many studies have leveraged the LSPR properties of Au NPs to detect various viral proteins,^17,18^ bacteria,^19,20^ and other disease-related organisms.^21^

The Langmuir–Blodgett (LB) technique has played a significant role in handling the self-assembly of biomaterials or nanoparticles.^22^ Assembling ligands naturally float on the solvent surface due to the surface tension of the water underneath, which allows for the monolayered assembly of ligands. These advantages make LB films invaluable in the fabrication of various monolayers of materials like Pt nanocrystals,^23^ graphene oxides,^24,25^ iron oxides,^26^ and many other organic films.^22^ The proper application of the LB technique in the fabrication of Au NP films could result in monolayered Au NPs with well-controlled thickness and superior properties.^27–29^ These LB films used in combination with LSPR as the transduction are expected to exhibit superior properties for sensing applications. Such characteristics could be applied in the detection of BoNT with promising facile detection, high sensitivity, elimination of animal testing, and compatibility with portable devices. In this study, a plasmonic nanosensor based on LB films of Au NPs was prepared. The LB films were examined in terms of surface pressure to elucidate the optimal films, which were then used to detect BoNT.

## Experimental

### Materials

The chemicals used in this study were sourced from Sigma-Aldrich including gold(iii) chloride trihydrate (HAuCl_4_·3H_2_O), tetraoctylammonium bromide (TOAB, ≥99%), sodium tetrahydridoborate (NaBH_4_), toluene, 1-aminododecane (DDA), ethyl alcohol (anhydrous), formic acid dimethylamide (DMF, anhydrous, 99%) 11-mercaptoundecaoic (11-MUA, 97%), *N*-(3-dimethylaminopropyl)-*N*′-ethylcarbodiimide hydrochloride (EDC), *N*-hydroxysuccinimide (NHS), hydroxylamine hydrochloride (NH_2_OH·HCl), and ammonia aqueous (NH_4_OH).

Botulinum neurotoxin type A toxoid from *Clostridium botulinum* (BoNT) and anti-botulinum neurotoxin type A (Chicken lgY) (Anti-BoNT) were obtained from List Labs, biotin conjugate kit was obtained from Abcam. Streptavidin was obtained from Thermo Scientific, and PET substrate were purchased from Sigma Aldrich, respectively. Deionized (DI) water (18.2 MΩ cm) was used in all of the experiments.

### Preparation of gold nanoparticles (Au NPs) and Langmuir–Blodgett (LB) films of the ligand-exchanged Au NPs

Au NPs of different sizes were synthesized using dodecylamine (DDA) as capping ligands through a phase transfer method as described in our previous studies.^24,30^ Briefly, 335 mg of HAuCl_4_ was first dissolved deionized water (30 ml). The resulting solution was combined with TOAB (2.2 g) using toluene solvent (80 ml) and vigorously mixed for a period of 1 hour. The floating organic phase was then collected, and 170 mg of DDA was introduced as capping ligands. Next, a solution made up of NaBH_4_ (380 mg) in 25 ml of DI was quickly added, which was then followed by stirring for an additional 3 hours. Again, the organic phase was then gathered and subjected to rotary evaporation for a period of 20 minutes. Ethanol was added to the sample in a quantity of 400 ml, and the solution was left to sit at a temperature of −17 °C overnight. Finally, filtration was conducted using 1 μm nylon membranes, and the residues were thoroughly rinsed with ethanol. The resulting Au NPs were subjected to a ligand-exchanging reaction with 11-MUA, which resulted in 11-MUA-capped Au NPs.

**Fig. 1:**
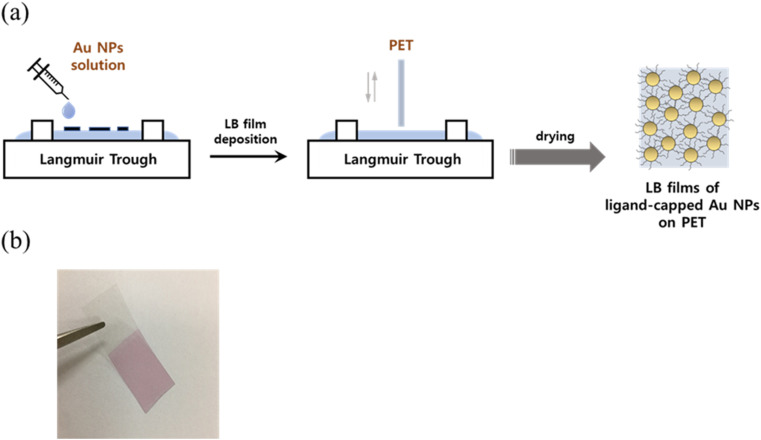
(a) Schematic illustration of fabrication of LB films of the ligand-capped Au NPs, and (b) optical image of the LB films deposited on PET.

Langmuir–Blodgett (LB) technique was utilized to deposit 11-MUA-capped Au NPs onto a PET film substrate as the procedure shown in Fig. 1a. In details, the PET film was thoroughly cleaned by subjecting it to a sonication bath containing acetone and DI water for 30 minutes, and then dried at 60 °C before further use. The LB technique involved spreading 300 μl of Au NPs onto DI water subphase, and compressing it at a speed of 10 mm min^−1^, with the trough open to air for 30 minutes. The PET substrate was then vertically withdrawn from the trough at 2 mm min^−1^ after reaching the expected surface pressure (35, 40 & 45 mN m^−1^), leading to the transfer of the deposited Au NPs films onto the PET film. The resulting LB films of Au NPs were subsequently air-dried under room conditions before use (Fig. 1b).

### Characterization

The dimensions of the MUA-capped Au NPs were analyzed with the use of high-resolution transmission electron microscopy (HR-TEM), specifically the JEM-300F model manufactured by JEOL. Additionally, the morphology of the LB films was assessed through field emission scanning electron microscopy (FE-SEM) using the JSM-7100F model from JEOL.

### Detection procedure

To activate the carboxyl groups on the 11-MUA molecules, the LB films of Au NPs LB film immersed in a mixed aqueous solution of NHS (0.05 M) and EDC (0.2 M) in a 1 : 1 volume ratio. After 30 minutes, the LB film was then immersed in a streptavidin solution in PBS buffer (2.2 μg ml^−1^) for immobilization. The LB film was next treated with NH_2_OH·HCl (0.5 M) for 1 hour, rinsed with water, and immersed in an anti-BoNT–biotin conjugate solution in PBS buffer (2 μg ml^−1^) for 1 hour. Previously, to prepare the antibody–biotin conjugate, biotin modifier reagent was added to the antibody, and the mixture was pipetted onto the lyophilized material with biotin quencher. The antibody-immobilized LB film was then immersed in the BoNT antigen solution for 1 hour at different concentrations, prepared by adding 1% NH_4_OH and diluting to 1 mg ml^−1^ with PBS buffer. To prevent unexpected bindings, the LB film was rinsed with the equivalent buffer/solvent before and after each immobilization and thoroughly with DI water before LSPR measurement. Using a UV-Vis machine (UH 3500, Hitachi), visible light in 450 nm to 800 nm wavelength range was supplied to measure extinction change and/or wavelength-shift, the LSPR signal, with bare PET film used as the reference substrate. All measurements were conducted in ambient conditions.

## Results and discussion

**Fig. 2:**
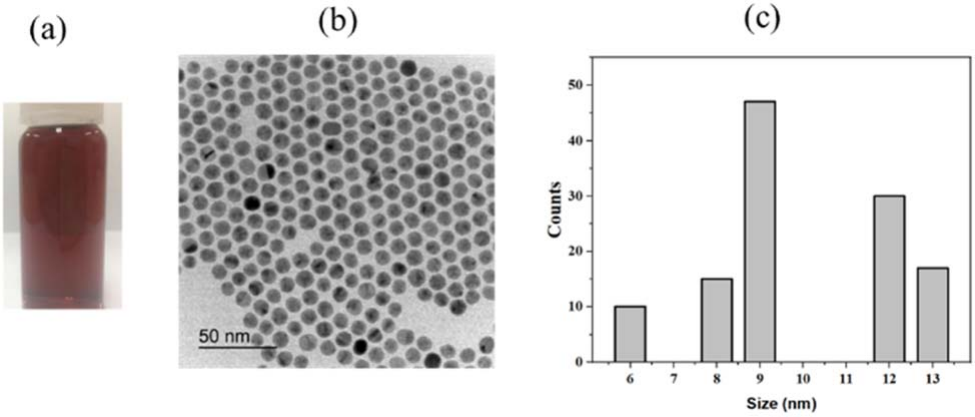
The as prepared Au NPs, (a) dispersion using hexane as solvent, (b) TEM image, and (c) size distribution.

The plasmonic material, ligand-capped Au NPs, was synthesized and characterized using HR-TEM as shown in Fig. 2. It can be observed that a good dispersion of the Au NPs in DMF was achieved, an essential requirement for the preparation of LB films on the water subphase.^31,32^ The Au NPs, exhibiting a hexagonal shape, present a wide size distribution ranging from 6 nm to 13 nm. This diversity in size enhances the range of the evanescent field generated by the Au NPs.^21,33^ In these ligand-exchanged Au NPs, the core consists of inorganic solids, while the shell is made up of organic ligands that contain functional groups – carboxyl groups, in this case. This composition imparts amphiphilic properties to the Au NPs. By leveraging the LB technique, monolayered films of the Au NPs were spread on the subphase, with each gold particle situated adjacent to the next, connected *via* hydrogen bonding from the carboxyl groups.^9,21^ The interconnecting ligands play a critical role in shaping the layer morphology and preventing the Au NPs from overlapping, which could result in severe aggregation. The transfer of the self-assembled layer was easily achieved by lifting the PET substrate out of the water. The Au NPs monolayer exhibited a high affinity for the PET surface, which also offered benefits in preserving the monolayer structure without altering the arrangement of the Au NPs in the layer. At this point, the LB technique has been proven to be a facile and effective approach for handling such a core–shell structure that is hard to achieve with other deposition techniques.

**Fig. 3:**
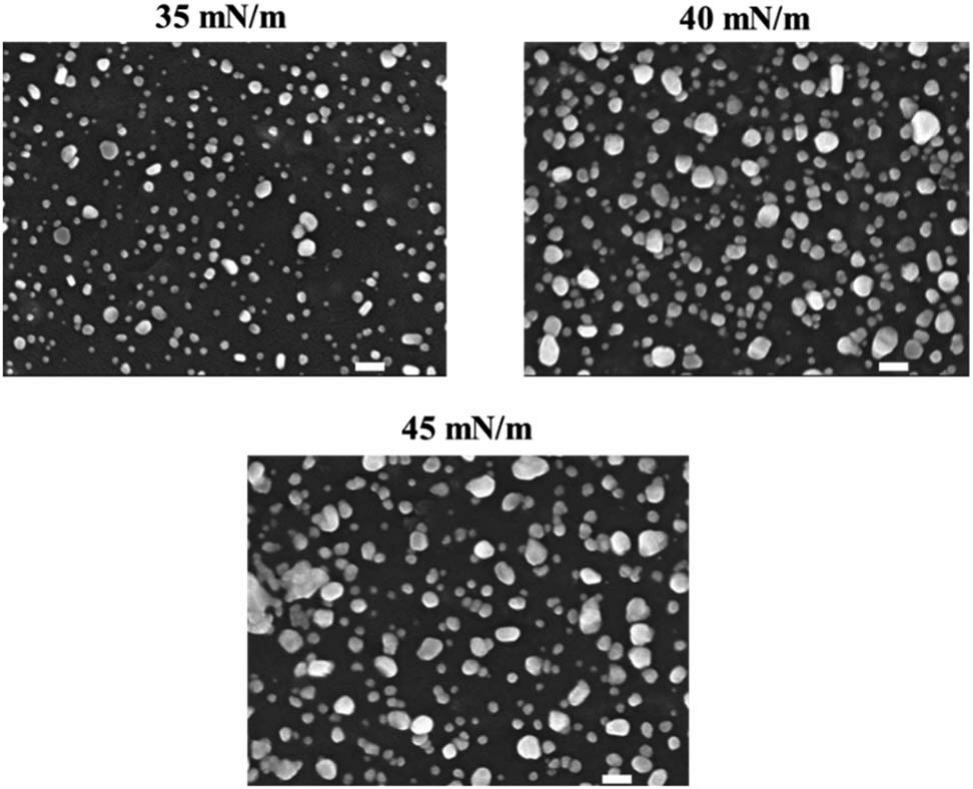
SEM images of the LB film fabricated at different values of surface pressure. The scale bar is 100 nm.

The Au NPs monolayer was transferred at different surface pressures of 35, 40, and 45 mN m^−1^, and the resulting LB films were characterized by FE-SEM as shown in Fig. 3. It is clear that the higher the surface pressure applied, the larger the gold clusters formed. As expected, the increase in surface pressure was known to narrow the separation between adjacent Au NPs, causing quick merging and resulting in the formation of enlarged clusters of the Au NPs. In the LB films transferred at 40 and 45 mN m^−1^, the Au NPs were observed in the form of large clusters rather than as single Au NPs. The plasmonic properties of these three different types of LB films were then investigated. As mentioned earlier, the LSPR that occurs around the Au NPs can be used as signal indicators to monitor changes in the refractive index of the surroundings. This is strongly dependent on the chemical composition of the layers deposited on the gold surface.^34,35^ In other words, each deposition on the Au NPs can be distinguished using the LSPR signal.

**Fig. 4:**
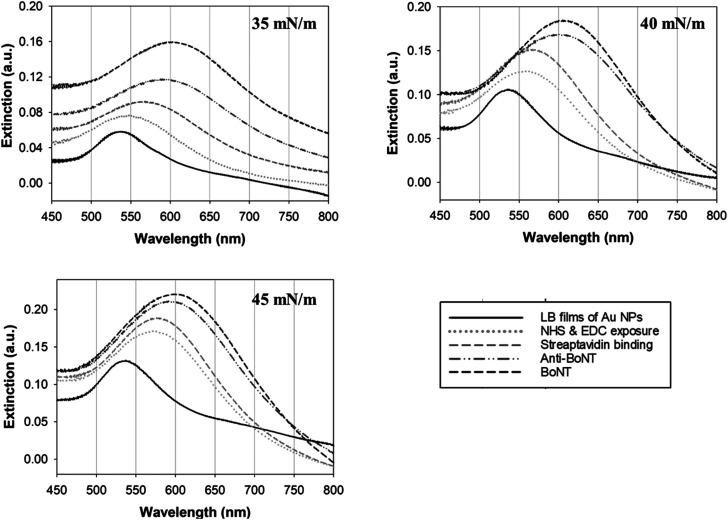
LSPR signals obtained from the LB film fabricated at different values of surface pressure.

**Fig. 5:**
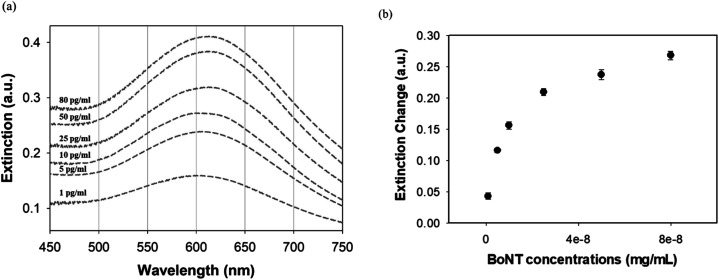
(a) LSPR signals with different concentrations of BoNT using LB films deposited at 35 mN m^−1^, and (b) dependency of extinction change on BoNT concentrations.

In this study, changes in UV-vis absorbance spectra were monitored upon the deposition of each layer. There are two types of plasmonic responses: (i) wavelength-shift (WS) observed at the maximum absorbance peak, recorded before and after deposition; (ii) extinction change (EC), or absorbance intensity, measured concurrently with the wavelength-shift. As shown in Fig. 4, when the core–shell Au NPs were deposited with ligands different from the existing ones, both WS and EC were simultaneously induced. This was observed during the sequential deposition of the following ligands: NHS & EDC, streptavidin, anti-BoNT, and BoNT. Meanwhile, the addition of the same type of materials to the shell was observed to cause EC without WS. This was seen to apply to all types of ligands used in this detection procedure. Apparently, the LB films, obtained at different surface pressures and corresponding cluster sizes, exhibited various LSPR behaviors. The sensing signal is attributed to the WS and/or EC upon the addition of the BoNT layer. The films transferred at 35 mN m^−1^ produced the most significant WS and EC compared to those at 40 & 45 mN m^−1^. In particular, there were WS of 11 nm along with EC of 0.043 a.u. observed in the UV-vis absorbance spectra as shown in Fig. 4a. Meanwhile, as shown in Fig. 4b and c, the sensing signals were much narrower in terms of both WS and EC. This was due to the formation of enlarged clusters of Au NPs resulting from the compression, as discussed in the FE-SEM results. This phenomenon is known as the dominant cause of reducing the characteristic decay length of the electric field, *l*_d_.^36,37^ The decrease in *l*_d_ is directly related to the sensing capacity of the LB films because events outside of *l*_d_ are known to no longer affect the LSPR signal. This is somewhat consistent with the UV-vis results: the LSPR signals were seen to weaken when the outer layers of anti-BoNT and BoNT were added. However, the initial LSPR signals were still strong for the first two layers of Au NPs and NHS & EDC. In addition, for the LB films transferred as 35 mN m^−1^, anti-BoNT immobilization induced LSPR peak at 588 nm with extinction of 0.117 a.u, meanwhile BoNT immobilization exhibited LSPR peak at 605 nm with extinction of 0.159 a.u. This obvious WS and EC could then be used as sensing signals. Given the superior characteristics mentioned above, the LB films transferred at 35 mN m^−1^ were selected for immersion into BoNT solutions of different concentrations, and then characterized by UV-vis absorbance. As shown in Fig. 5, upon the immobilization of BoNT, the LB films produced extinction peaks located around 605 nm for all concentrations. As the concentration increased, so did the extinction. It is clear that it is feasible to use the LSPR signal, or EC, to distinguish different ranges of BoNT concentrations. The dependence of LSPR responses on BoNT concentrations is depicted in Fig. 5b, and it demonstrates non-linear behavior, consistent with findings from previous studies as the equation below:^37^

**Fig. 6:**
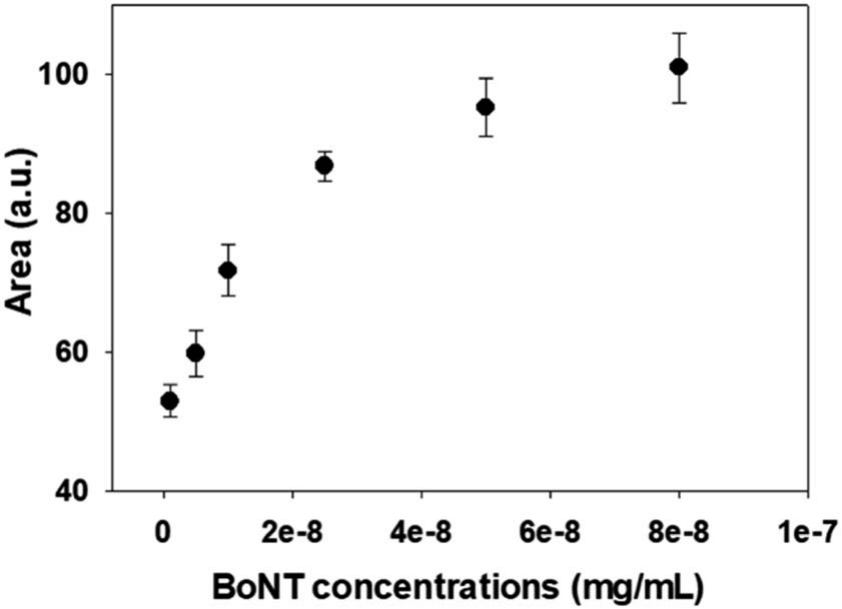
Dependency of area under curves with BoNT concentrations.

1 Δ*R* = *m*(*n*_BoNT_ − *n*_N_2__)e^−2*d*_SAM_/*l*_d_^(1 − e^−2*d*_BoNT_/*l*_d_^)

where Δ*R* is the plasmonic response, *m* is the refractive index sensitivity of the plasmonic particles, *d*_BoNT_ is the thickness of the adsorbed BoNT and its refractive index, *n*_BoNT_, *n*_N_2__ represents the bulk refractive index of the external medium, and *d*_SAM_ is the total thickness of SAM layers. As is clearly shown in eqn (1), the response exhibits inverse exponential growth, which strongly aligns with the obtained results for BoNT. The plasmonic response is displayed as a steep change in the first few ranges of concentration, followed by a gradual appearance of a plateau. The addition of BoNT with different concentrations induced increment in the background, so the areas under curves were also provided to clarify the changes in LSPR signal as shown in Fig. 6. The changes of the areas were observed to be consistent with extinction changes, which can also be used as another sensing signal.

## Conclusions

The sensor fabricated from LB films of Au NPs was successfully used to detect BoNT in a PBS buffer, with a range of concentrations from 1 pg ml^−1^ to 80 pg ml^−1^. The use of the LB technique conferred tremendous advantages in controlling the morphology of the Au NPs. Simply changing the surface pressure resulted in different monolayers of Au NPs. Clusters of Au NPs with enlarged sizes were found to be less sensitive to the binding events occurring on the LB films, which could be attributed to a significantly reduced characteristic decay length. The plasmonic response was confirmed to exhibit an inverse exponential growth against the BoNT concentrations. The limit of detection was 1 pg ml^−1^ using LB films transferred at 35 mN m^−1^, a detection range as small as the lethal dose mentioned earlier. These exploratory results show promising potential for employing visible light in the detection of BoNT in real food samples.

## Author contributions

Ly & Nguyen designed experiment procedure. Nguyen conducted experiment and collected data. Ly analysed data along with Nguyen. Ly compiled results and wrote manuscript.

## Conflicts of interest

There are no conflicts to declare.

## Supplementary Material
